# GSCI: A Generative and Sparse Compressed Sensing Imputation Framework for Single-Cell RNA-Sequencing Dropout Recovery

**DOI:** 10.34133/csbj.0124

**Published:** 2026-07-23

**Authors:** Yan Tian, Meng Li, Bo Han, Jun Zhang, Tao You, Ruihao Xin, Xin Feng

**Affiliations:** ^1^ Jilin City Hospital of Chemical Industry, Jilin 130000, P. R. China.; ^2^ School of Artificial Intelligence, Jilin University of Chemical Technology, Jilin 130000, P. R. China.; ^3^ College of Electrical and Control Engineering, Jilin University of Chemical Technology, Jilin 130000, P. R. China.

## Abstract

Single-cell RNA-sequencing data are inherently sparse and high-dimensional, with dropout events introducing a large number of missing values that hinder downstream analyses. To address the dual challenges of data imputation and dimensionality reduction, we propose an integrated recovery framework that combines probabilistic modeling with sparse optimization. The proposed method captures latent distribution patterns underlying gene expression, enabling the reconstruction of nonlinear dependencies among genes. Furthermore, sparsity constraints and heuristic search strategies are employed to enhance recovery accuracy, particularly in regions with low expression, while maintaining global expression consistency. We evaluate the framework on in-domain breast-cancer cohorts, on mask-augmented datasets with prescribed masking ratios and known references, and on 5 heterogeneous external datasets spanning distinct protocols and scales; across these settings, the method outperforms representative baselines in reconstruction error, structural fidelity, and recovery of biologically critical genes. These results highlight the effectiveness of jointly modeling distributional structure and sparsity for the reliable restoration of single-cell expression data.

## Graphical Abstract

**Figure:**
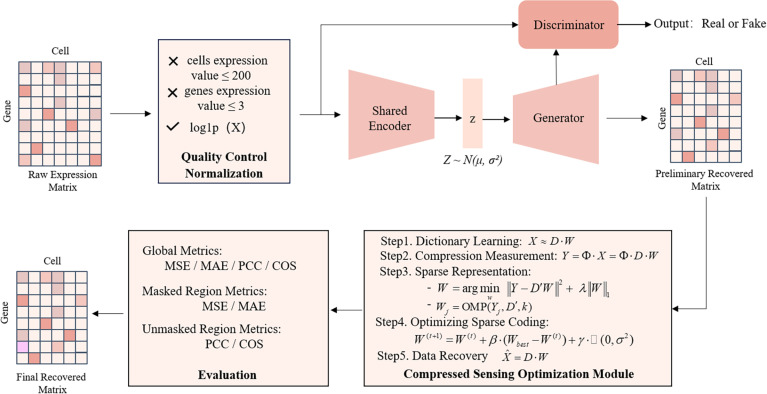

## Introduction

Single-cell RNA sequencing (scRNA-seq) has become indispensable for resolving cellular heterogeneity, reconstructing developmental programs, and probing disease mechanisms at single-cell resolution [1–3]. By profiling thousands of genes per cell, scRNA-seq enables the discovery of rare states and disease-relevant biomarkers and yields refined views of tissue microenvironments [4–6]. However, the data remain computationally challenging: Measurements are noisy, highly sparse due to dropout events, and extremely high-dimensional relative to the number of cells (“large-P, small-N”) [7–9]. Dropouts—false zeros induced by limited capture efficiency and sequencing depth—distort cell–cell and gene–gene relationships and can mislead clustering, trajectory inference, and differential expression (DE) analyses [10,11].

Over the past years, a diverse repertoire of methods has sought to mitigate dropout and improve downstream interpretability. Graph-diffusion approaches (e.g., Markov affinity-based graph imputation of cells [MAGIC]) propagate signals along cell neighborhoods to denoise expression [12], while deep autoencoders and variational models (e.g., deep count autoencoder [DCA] and single-cell variational inference [scVI]) learn nonlinear latent representations for reconstruction [13,14]; statistical/matrix-factorization models (e.g., scImpute, SAVER, and ALRA) impose distributional or low-rank assumptions [15–17] and gene-centric regressors (e.g., DeepImpute) leverage local gene dependencies [18]. More recently, adversarial generators—exemplified by single-cell imputation generative adversarial networks (sciGANs) and single-cell multimodal generative adversarial network (scMultiGAN)—improve distributional fidelity by learning data manifolds with generative adversarial network (GAN)-based objectives [19,20]. Although these methods have substantially improved scRNA-seq denoising and imputation, important trade-offs remain in balancing distribution-aware recovery, explicit sparsity control, and preservation of biologically meaningful structure. In particular, some approaches favor manifold smoothing but may oversmooth subtle variation, whereas others preserve local structure better but provide limited control over sparse reconstruction behavior. Moreover, real scRNA-seq datasets generally lack true expression ground truth, so reconstruction-style metrics alone may not fully reflect downstream biological usefulness. Classical linear dimensionality-reduction techniques—principal component analysis (PCA), independent component analysis (ICA), and nonnegative matrix factorization (NMF)—are typically applied post hoc to obtain low-dimensional embeddings [21–23]; while efficient, their linear/low-rank assumptions can underfit the nonlinear, zero-inflated geometry of single-cell manifolds. At the same time, recent scRNA-seq recovery models have increasingly combined latent representation learning with denoising or structured regularization to varying extents. More recent recovery frameworks have further extended this trend by introducing stronger sparsity-aware or hybrid reconstruction strategies [24], indicating that simple “joint modeling” is no longer, by itself, a sufficient novelty claim. In this context, the contribution of the present study is not to claim the first hybrid framework but to develop a coordinated 2-stage recovery strategy in which structure-guided generative pre-recovery is followed by compressed-sensing-based sparse refinement under a zero-aware objective. These considerations motivate a 2-stage recovery strategy in which distribution-aware denoising and sparsity-regularized reconstruction are combined within a unified framework.

To this end, we propose the generative and sparse compressed sensing imputation (GSCI)—a framework that couples a structure-guided generative module (SGM) with a compressed-sensing-driven optimization reconstruction module (CSO). A zero-aware objective explicitly shapes recovery differently in zero-inflated and expressed regions, mitigating oversmoothing and adversarial hallucination while preserving high-expression structures. By coupling generative pre-recovery with sparse refinement, GSCI aims to produce reconstructions that are both structurally coherent and suitable for downstream single-cell analyses.

We formalize the problem setting and the resulting optimization in the next section (Problem formulation), where we detail the learning objective, the zero-aware design, and the sparsity-regularized reconstruction mechanism used in our framework.

Contributions. Our work makes 2 contributions:

1. Coordinated 2-stage recovery framework. We develop the GSCI, which couples an SGM with a sparsity-governed compressed-sensing module to jointly perform dropout imputation and dimensionality reduction within a single framework, yielding compact, interpretable representations.
2. Zero-aware nonlinear–sparse modeling. We design a zero-aware objective that treats zero-inflated and expressed regions differently and incorporates dictionary-level sparsity, curbing over-imputation while preserving salient expression structure.

## Problem Formulation

Let $X\in {\mathbb{R}}_{\geq 0}^{g\times n}$ denote the unknown true expression matrix after standard normalization (and log(1+·) when stated). We observe $O\in R^{g\times n}$ with a binary mask $\Omega \in {\left\{0,\;1\right\}}^{g\times n}$:

$$O=\Omega ⊙X+\xi_{\mathrm{obs}},$$ (1)

where ⊙ is the Hadamard product and ε denotes residual technical noise. We assume a missing-not-at-random dropout mechanism, i.e., the detection probability depends on the underlying abundance, with $\mathrm{Pr}\left(\Omega_{\mathrm{ij}=1}|\;X_{\mathrm{ij}}\right)$ increasing with $X_{\mathrm{ij}}$.

Goals. Recover $\hat{X}$ that preserves expressed signals without over-imputing truly absent genes, and learn a compact embedding $Z\in {\mathbb{R}}^{d\times n}\left(d\ll g\right)$ suitable for downstream tasks. We formalize a method-agnostic objective:

$$\min_{\hat{X},Z}L_{\mathrm{rec}}\left(\hat{X};\;O,\;\Omega \right)+R\left(\hat{X},\;Z\right),$$ (2)

where $L_{\mathrm{rec}}$ can be zero-aware—assigning different weights to dropped versus observed regions—and *R* promotes compactness, stability and scalability.

Transition to methods. In the next section, we present one concrete instantiation of Eq. (2) that couples distribution-aware generative denoising with sparsity-governed reconstruction to realize these desiderata.

## Methodology

### Theoretical basis

#### Variational autoencoder

The variational autoencoder (VAE) is a probabilistic latent-variable model grounded in the concept of generative modeling. Its primary goal is to construct a probabilistic distribution in a latent space, enabling the effective compression and reconstruction of high-dimensional data. Unlike traditional autoencoders, VAE introduces the concept of variational inference during the encoding process, mapping the input to a conditional distribution rather than a fixed code. This endows the model with stronger generative and generalization capabilities [25,26].

Here, $x\in {\mathbb{R}}_{\geq 0}^{g}$ denotes a single-cell expression vector. In terms of architecture, the VAE consists of an encoder, a latent sampling module, and a decoder. The encoder maps 𝑥 to the parameters of a latent distribution, typically a mean $\mu \left(x\right)$ and a standard deviation vector $\sigma \left(x\right)$, thereby parameterizing $\begin{array}{c} q_{\phi }\left(z|x\right)=N\left(z;\;\mu_{\phi }\left(x\right),\;\mathrm{diag}\left(\sigma_{\phi }^{2}\left(x\right)\right)\right) \end{array}$.

To ensure differentiability, the reparameterization trick is applied: $z=\mu_{\phi }\left(x\right)+\sigma_{\phi }\left(x\right)⊙\xi_{\mathrm{vae}},\xi_{\mathrm{vae}}∼N\left(0,\;I\right)$.

The decoder specifies a likelihood $p_{\theta }\left(x|z\right)$ (e.g., Gaussian for normalized/log-transformed data or negative binomial/Poisson for raw counts). To accurately reconstruct the input while learning a well-structured latent space, training maximizes the evidence lower bound, which combines a reconstruction term with a Kullback–Leibler (KL) regularizer:

$$\begin{array}{c} L_{\text{ELBO}}\left(x\right)=E_{q_{\phi }\left(z|x\right)}\left[\log p_{\theta }\left(x|z\right)\right]-\mathrm{KL}\left(q_{\phi }\left(z|x\right)\left\|p\left(z\right)\right.\right), \end{array}$$ (3)

where $E_{q_{\phi }\left(z|x\right)}$ denotes expectation under $q_{\phi }\left(z|x\right)$ and $p\left(z\right)=N\left(0,\;I\right)$.

This objective not only maximizes reconstruction fidelity but also guides the encoder to learn a continuous and regularized latent distribution. In this study, the VAE serves to capture the low-dimensional latent structure of single-cell expression data and to provide a preliminary reconstruction that facilitates dropout recovery. Implementation details of our instantiation are provided in the “Methodological framework and module design” section.

#### Generative adversarial network

The GAN is a representative framework for generative modeling, consisting of 2 components: a generator and a discriminator. Through adversarial training between these 2 networks, the model learns to capture the underlying data distribution and synthesize samples from random noise [27,28]. The generator maps noise to samples that resemble real data, while the discriminator attempts to distinguish between real and generated samples [29]. This interplay encourages the model to capture intrinsic structure of the data distribution [30].

In a typical GAN, the generator receives a random noise vector $z∼p_{z}\left(z\right)$ sampled from a latent prior and transforms it through a series of nonlinear mappings to produce synthetic samples $G\left(z\right)$. Real samples $x∼p_{\text{data}}\left(x\right)$ and generated samples $G\left(z\right)$ are fed to the discriminator $D_{\mathrm{gan}}\left(\cdot \right)$, which outputs the probability that an input is real. Training is formulated as the following minimax game:

$$\begin{array}{c} \min_{G}\max_{D_{\mathrm{gan}}}\;E_{x∼p_{\text{data}}\left(x\right)}\left[\log D_{\mathrm{gan}}\left(x\right)\right]\\ +E_{z∼p_{z}\left(z\right)}\left[\log\left(1-D_{\mathrm{gan}}\left(G\left(z\right)\right)\right)\right], \end{array}$$ (4)

where G denotes the generator and $D_{\mathrm{gan}}$ is the discriminator. The first term increases the discriminator’s output on real samples, while the second term encourages low discriminator confidence on generated ones; conversely, optimizing G drives $D_{\mathrm{gan}}\left(G\left(z\right)\right)$ upward, pushing the model distribution toward the empirical data distribution [31].

In our framework, adversarial learning is used as a distribution-matching regularizer, and the concrete instantiation appears in the “Methodological framework and module design” section.

#### Compressed sensing

Compressed sensing is a theoretical framework designed to reconstruct high-dimensional sparse signals from a small number of linear observations. It has been widely applied in fields such as image reconstruction, seismic detection, and wireless sensing [32]. The core assumption is that if a signal has a sparse representation in some transformation domain, it can be recovered from data sampled at a rate much lower than the Nyquist rate through linear observations and sparse optimization [33,34].

Compressed sensing has been successfully applied in various fields, including image processing, wireless sensing, and seismic exploration, to recover high-quality signals from undersampled data [35,36]. Its key principle lies in utilizing the sparse prior of the signal to achieve high-precision recovery from data sampled at a rate much lower than the Nyquist rate [37]. This characteristic aligns well with the dropout phenomenon in single-cell transcriptomic data. In scRNA-seq data, gene expression matrices are inherently high-dimensional and sparse, and dropout noise can be viewed as a “partial observation” of sparse signals [38]. Therefore, compressed-sensing theory provides a mathematical foundation for recovering the complete gene expression profile from sparse, noisy observations.

Formally, let $x\in {\mathbb{R}}^{g}$ denote a single-cell expression vector that is sparse in a dictionary $\Psi \in {\mathbb{R}}^{g\times d},i.e.,x=\Psi u$ with $u\in {\mathbb{R}}^{d}$ and ${\left\|u\right\|}_{0}\ll d$. Linear measurements are modeled as

$$y=\Phi x+\xi_{\mathrm{cs}},\;\Phi \in {\mathbb{R}}^{m\times g},\;m\ll g,$$ (5)

where Φ is the measurement operator and $\xi_{\mathrm{cs}}$ denotes additive noise. A common recovery formulation estimates a sparse code *u* via

$$\min_{u}\frac{1}{2}{\left\|y-\Phi \Psi u\right\|}_{2}^{2}+\lambda {\left\|u\right\|}_{1},\;\hat{x}=\Psi \hat{u},$$ (6)

or, equivalently, $\min_{u}{\left\|u\right\|}_{1}s.t.{\left\|y-\Phi \Psi u\right\|}_{2}\leq \varepsilon$. Solvers include convex relaxations (LASSO-type), greedy methods (e.g., orthogonal matching pursuit, OMP), and first-order forward–backward schemes; the choice of λ and the dictionary Ψ trades off accuracy and computation.

**Fig. 1.**
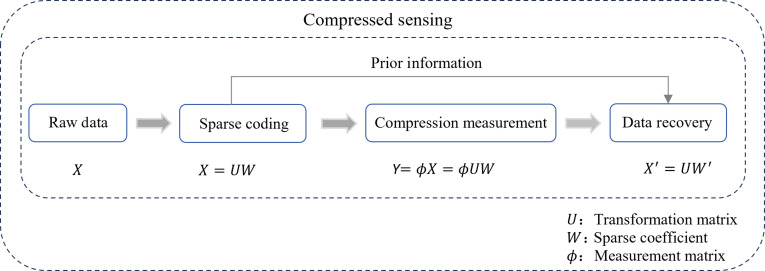
Compressed sensing principle diagram.

To better understand this process, Fig. 1 illustrates the compressed-sensing workflow, from sparse coding of the original data to compressed measurements and data recovery.

Remark. In the “Methodological framework and module design” section, we instantiate the compressed sensing framework with a learned dictionary *D* and coefficients *W* (replacing Ψ, *u*), yielding a compressed-sensing-driven optimization coupled with the generative module.

#### Nature-inspired optimization algorithms

Nature-inspired optimization algorithms emulate self-organizing mechanisms observed in biological collectives or physical phenomena to solve complex nonlinear optimization problems [39,40]. The fundamental idea is to transform the optimization objective into a fitness function and approximate the better individual through the cooperation and competition among individuals within a population [41].

In compressed-sensing-related reconstruction tasks, conventional convex optimization methods (e.g., LASSO) and greedy algorithms (e.g., OMP) remain important and effective tools for sparse estimation [42,43]. In the present study, we do not introduce a nature-inspired optimizer as a replacement for these standard solvers. Instead, it is incorporated into the reconstruction framework as a population-based optimization strategy to guide the refinement of candidate sparse solutions under the task-specific objective. For instance, genetic algorithms generate diverse candidate solutions through crossover and mutation operations, while particle swarm optimization guides the search using both individual and global historical best positions [44,45]. Such methods are attractive because they can explore complex search spaces in parallel and provide a flexible mechanism for structured refinement when reconstruction objectives go beyond standard sparse coding alone. At the same time, population-based algorithms may introduce additional computational cost and require careful control of update parameters, which should be considered when they are applied in high-dimensional settings [46–48].

The Ivy Algorithm, inspired by the growth pattern of ivy plants, simulates the biological mechanism by which a plant selects the optimal neighboring path for self-optimization, thereby enhancing both global search and local exploitation capabilities [49]. The core idea of the algorithm is that each updates its position by moving toward the most favorable neighbor, based on both the fitness and spatial location of surrounding individuals. To prevent convergence to local optima, the algorithm introduces a degree of randomness into the update process, thereby reinforcing its global exploration ability.

The individual update process in the Ivy Algorithm optimizes the search trajectory by iteratively adjusting the positions of individuals. Let the position of the *i*-th individual at iteration *t* be denoted as $p_{i}^{\left(t\right)}$, and the update rule is formulated as

$$P_{i}^{\left(t+1\right)}=p_{i}^{\left(t\right)}+\beta \cdot \left(p_{j}^{\left(t\right)}-p_{i}^{\left(t\right)}\right)+\gamma \cdot N\left(0,\;\sigma^{2}\right),$$ (7)

where $P_{i}^{\left(t\right)}$ denotes the position of the *i*-th individual (i.e., a candidate solution) while $P_{j}^{\left(t\right)}$ represents the position of the optimal neighbor, typically the individual with the lowest fitness. β denotes the growth factor, which controls the step size toward the optimal neighbor. γ represents the stochastic expansion factor that introduces random perturbations to enhance the diversity of exploration. $N\left(0,\;\sigma^{2}\right)$ corresponds to Gaussian noise, which increases search randomness and helps prevent the algorithm from converging to local optima.

The growth factor β is dynamically adjusted according to the distance between the individual and its optimal neighbor, as defined by the following equation:

$$\beta =\beta_{0}\cdot \exp\left(-\mu \cdot r_{\mathrm{ij}}^{2}\right),$$ (8)

where $\beta_{0}$ denotes the initial growth factor, which controls the initial movement speed of an individual toward its optimal neighbor, while μ represents the decay factor, which regulates the sensitivity of the growth factor to the distance. A larger μ implies a stronger response of the individual’s movement to the distance, favoring rapid exploration; whereas a smaller μ indicates a weaker response, favoring fine-grained local search. The Euclidean distance between individuals *i* and *j* is defined as

$$d_{\mathrm{ij}}=\sqrt{\sum_{k=1}^{n}{\left(P_{i}^{\left(t\right)}-P_{j}^{\left(t\right)}\right)}^{2}},$$ (9)

Fitness evaluation is a core component of the Ivy Algorithm. In the general Ivy formulation, the fitness value of each individual reflects the quality of its current position with respect to the adopted search objective. Individuals with lower fitness values are more attractive to others, thereby guiding the population toward better candidate solutions. The generic fitness evaluation is defined as

$$f\left(L_{i}\right)={\left\|P_{i}-P^{*}\right\|}^{2},$$ (10)

where *f*(*L_i_*​) denotes the fitness value of the *i*-th individual and P* represents the current optimal neighbor or best candidate in the population. A smaller fitness value indicates a better solution under the adopted search criterion. In our framework, this general Ivy mechanism is used to guide the refinement of sparse reconstruction within the CSO module, whereas the task-specific fitness function adopted for reconstruction is introduced later in the “Compressed-sensing-driven optimization reconstruction module” section.

### Methodological framework and module design

#### Overall architecture

**Fig. 2.**
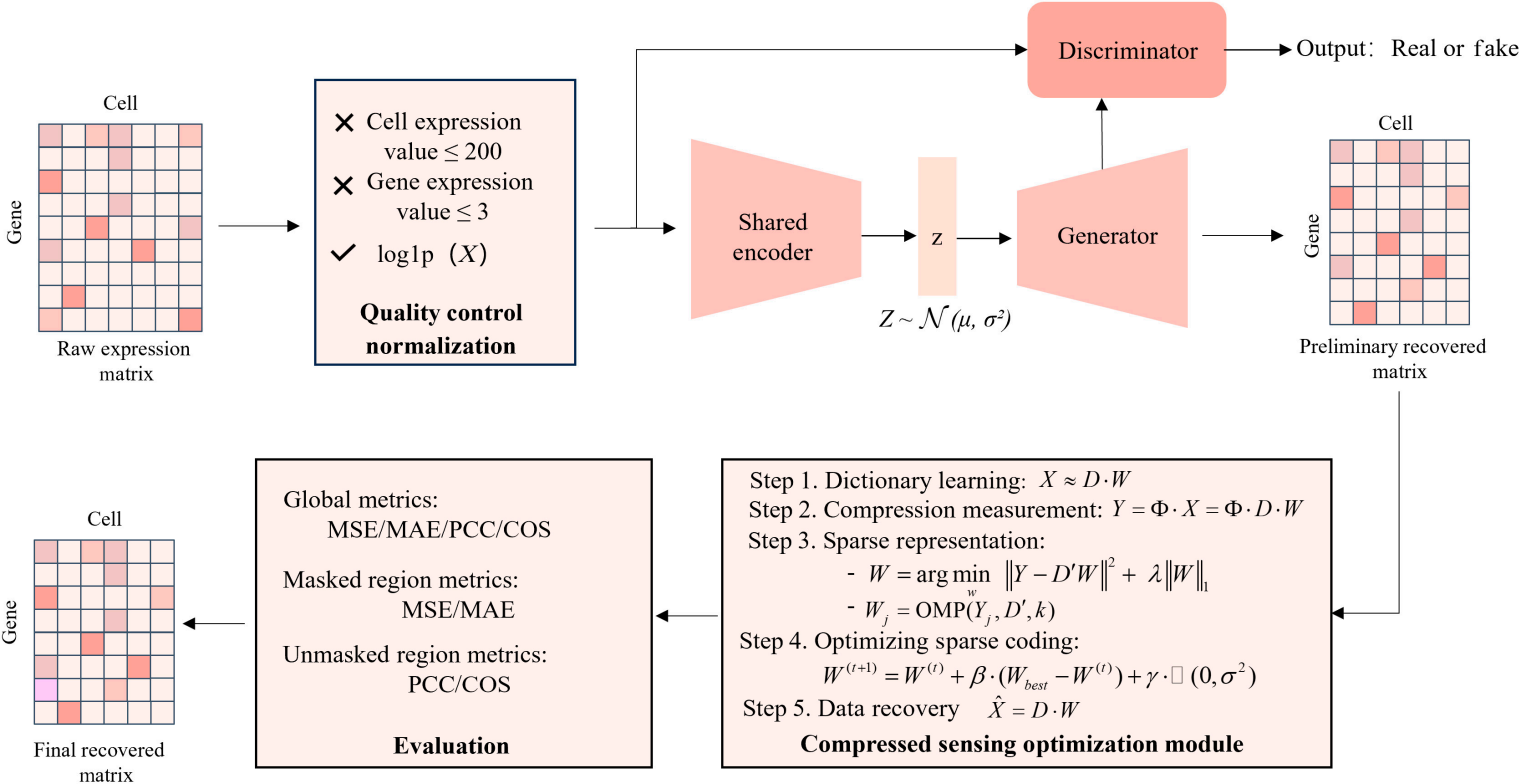
Generative and sparse compressed sensing imputation (GSCI) framework module structure diagram. MAE, mean absolute error; MSE, mean squared error; OMP, orthogonal matching pursuit; PCC, Pearson correlation coefficient; COS, cosine similarity.

To address the dropout phenomenon in scRNA-seq data while preserving both structural consistency and biological characteristics, we propose a 2-stage recovery framework named GSCI, which integrates generative modeling theory with sparse compressed sensing optimization. As illustrated in Fig. 2, the overall workflow is designed to progressively model the latent structure and sparse representation of gene expression data, thereby enabling the restoration of structural information and improving the accuracy of missing values.

(a) Data preprocessing

Following standard procedures, the raw single-cell expression matrix undergoes quality control and normalization. Cells with low expression (cell expression ≤ 200) and genes with low abundance (gene expression ≤ 3) are filtered out. Logarithmic normalization is then applied to stabilize the data distribution, providing a more precise expression pattern for downstream modeling.

(b) Structure-guided generative module

This module is built upon probabilistic generative modeling theory, guiding the model to recover the latent distributional characteristics of expression structures from the dropout-affected input matrix. The SGM consists of a shared encoder, a generator, and a discriminator. It jointly optimizes the reconstruction error and distributional consistency, enhancing the model’s capacity to learn gene expression in zero-inflated regions and enabling latent-space modeling of complex gene dependency structures.

(c) Compressed-sensing optimization module

Based on the preliminary reconstruction, the GSCI framework further introduces the CSO module to enhance structural fidelity and compression robustness. This module first extracts a fixed dictionary $D\in {\mathbb{R}}^{g\times d}$ via singular value decomposition, then constructs an observation matrix $\Phi \in {\mathbb{R}}^{m\times g}$ to obtain the compressed measurement matrix: $Y=\Phi \cdot \tilde{X}=\Phi \cdot D\cdot W$. On this basis, the sparse coefficients $W\in {\mathbb{R}}^{d\times n}$ are initialized using the OMP method and iteratively optimized by the Ivy Algorithm. Finally, the structurally stable and expression-fidelity reconstruction matrix is generated through $\tilde{X}=D\cdot W^{*}$.

(d) Evaluation and analysis

The model recovery results are comprehensively evaluated through multiple levels of metrics, including global indicators (mean squared error [MSE], mean absolute error [MAE], Pearson correlation coefficient [PCC], and cosine similarity [COS]), stratified evaluations for the zero-entry and non-zero-entry evaluations, as well as key gene recovery metrics. This ensures both the consistency of expression and the biological interpretability of the method.

#### Structure-guided generative module

To address the dropout issue caused by technical limitations in scRNA-seq data, the GSCI framework incorporates SGM in the first stage to construct a high-quality preliminary recovery expression matrix. This module integrates probabilistic generative modeling theory with an expression structure-guided mechanism. It aims to enhance the model’s ability to fit actual expression patterns and improve reconstruction performance in zero-inflated regions through latent-space modeling and data distribution reconstruction.

**Fig. 3.**
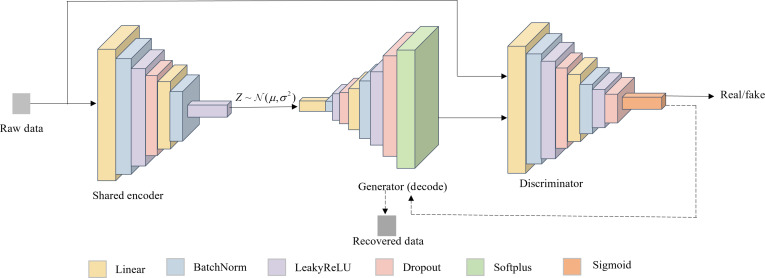
Structure-guided generative module.

The SGM consists of 3 core submodules: a shared encoder, a generator, and a discriminator. The processing workflow is illustrated in Fig. 3. Let the dropout-affected input matrix be denoted as $X\in {\mathbb{R}}^{g\times n}$, where *g* represents the number of genes and *n* is the number of cells. The encoder network maps each sample into a latent space, generating latent vectors $z\in {\mathbb{R}}^{d}$, where d≪g denotes the dimensionality of the latent space. The generation process of the latent variable *z* can be formulated using the encoding distribution of the VAE as

$$q\left(z|x\right)=N\left(\mu \left(x\right),\;\sigma^{2}\left(x\right)\right),$$ (11)

where $\mu \left(x\right)$ and $\sigma \left(x\right)$ represent the mean and variance outputs of the encoder, respectively, characterizing the distribution in the latent space.

To enable differentiable sampling, the reparameterization trick is employed to generate the latent variable:

$$z=\mu \left(x\right)+\sigma \left(x\right)\cdot \varepsilon ,\varepsilon \in N\left(0,\;1\right),$$ (12)

The latent variable *z* is then fed into the generator network to reconstruct the preliminary recovered expression matrix $\hat{X}\in {\mathbb{R}}^{g\times n}$:

$$\hat{X}=G\left(z\right),$$ (13)

where $G\left(\cdot \right)$ denotes the generator, which utilizes fully connected layers to map latent variables to high-dimensional data. The discriminator $D_{\mathrm{gan}}\left(\cdot \right)$ serves not only as a module for assessing sample authenticity but also as a feedback mechanism that guides the generator through adversarial training. This process enhances the biological consistency of reconstructed outputs in terms of expression statistics and structural priors. The output of the discriminator is

Dganx=Ρx∈Real, (14)

The SGM integrates latent representation learning and generative modeling through a shared encoder architecture. Guided by the latent space, it jointly optimizes the generation and discrimination objectives, while incorporating a dropout-aware mechanism to enhance sensitivity to missing regions and improve expression consistency. To enable joint training, the SGM utilizes a composite loss function comprising a variational reconstruction term and an adversarial generation term. The variational reconstruction loss is defined as

$$L_{\mathrm{VAE}}={Ε}_{q\left(z|x\right)}\left[{\left\|\hat{x}-x\right\|}^{2}\right]+\mathrm{KL}\left(q\left(z|x\right)\|p\left(z\right)\right),$$ (15)

where the first term represents the reconstruction error, while the second term corresponds to the KL divergence, and its mathematical expanded form is given by

$$\begin{array}{c} \mathrm{KL}\left(q\left(z|x\right)\|p\left(z\right)\right)=-\frac{1}{2}\sum_{j=1}^{d}\left(\mu_{j}^{2}+\sigma_{j}^{2}-\log\sigma_{j}^{2}-1\right), \end{array}$$ (16)

The GAN submodule adopts an adversarial loss, where the generator and discriminator engage in a minimax game by optimizing the following objective:

$$\begin{array}{c} \min_{G}\max_{D_{\mathrm{gan}}}{Ε}_{x∼P_{\text{data}}}\left[\log D_{\mathrm{gan}}\left(x\right)\right]+{Ε}_{z∼p\left(z\right)}\left[\log\left(1-D_{\mathrm{gan}}\left(G\left(z\right)\right)\right)\right], \end{array}$$ (17)

Finally, the overall training objective of the model is to optimize the following hybrid loss function jointly:

$$L_{\text{Hybrid}}=\alpha_{\mathrm{VAE}}\cdot L_{\mathrm{VAE}}+\beta_{\mathrm{GAN}}\cdot L_{\mathrm{GAN}},$$ (18)

where the hyperparameters $\alpha_{\mathrm{VAE}},\beta_{\mathrm{GAN}}$ control the relative contributions of the VAE and GAN branches, respectively, during joint training.

Additionally, to enhance the reconstruction quality on zero-entry positions, we incorporate a dropout-aware weighting mechanism into the reconstruction loss. This mechanism assigns higher weights to elements with zero values, thereby prioritizing their recovery during the optimization process. The weighted reconstruction loss is defined as follows:

$$\text{weight}\_\text{loss}=\sum_{i,j}\varpi_{\mathrm{ij}}\cdot {\left({\hat{x}}_{\mathrm{ij}}-x_{\mathrm{ij}}\right)}^{2},\varpi_{\mathrm{ij}}=\left\{\begin{array}{l} 3,x_{\mathrm{ij}}=0\\ 1,x_{\mathrm{ij}}\neq 0 \end{array}\right.,$$ (19)

This mechanism enhances the model’s sensitivity to zero-entry positions and serves as an effective optimization strategy for addressing technical missingness in single-cell sequencing data.

Figure 3 illustrates the overall architecture of the SGM, clearly depicting the entire pipeline: the dropout-affected input matrix is first mapped to a latent space via the encoder, then reconstructed by the generator, and finally refined under adversarial supervision provided by the discriminator. The encoder is shared between the VAE and GAN components to ensure unified sample representation learning, reduce parameter redundancy, and improve both the training stability and expression consistency of the SGM.

#### Compressed-sensing-driven optimization reconstruction module

**Fig. 4.**
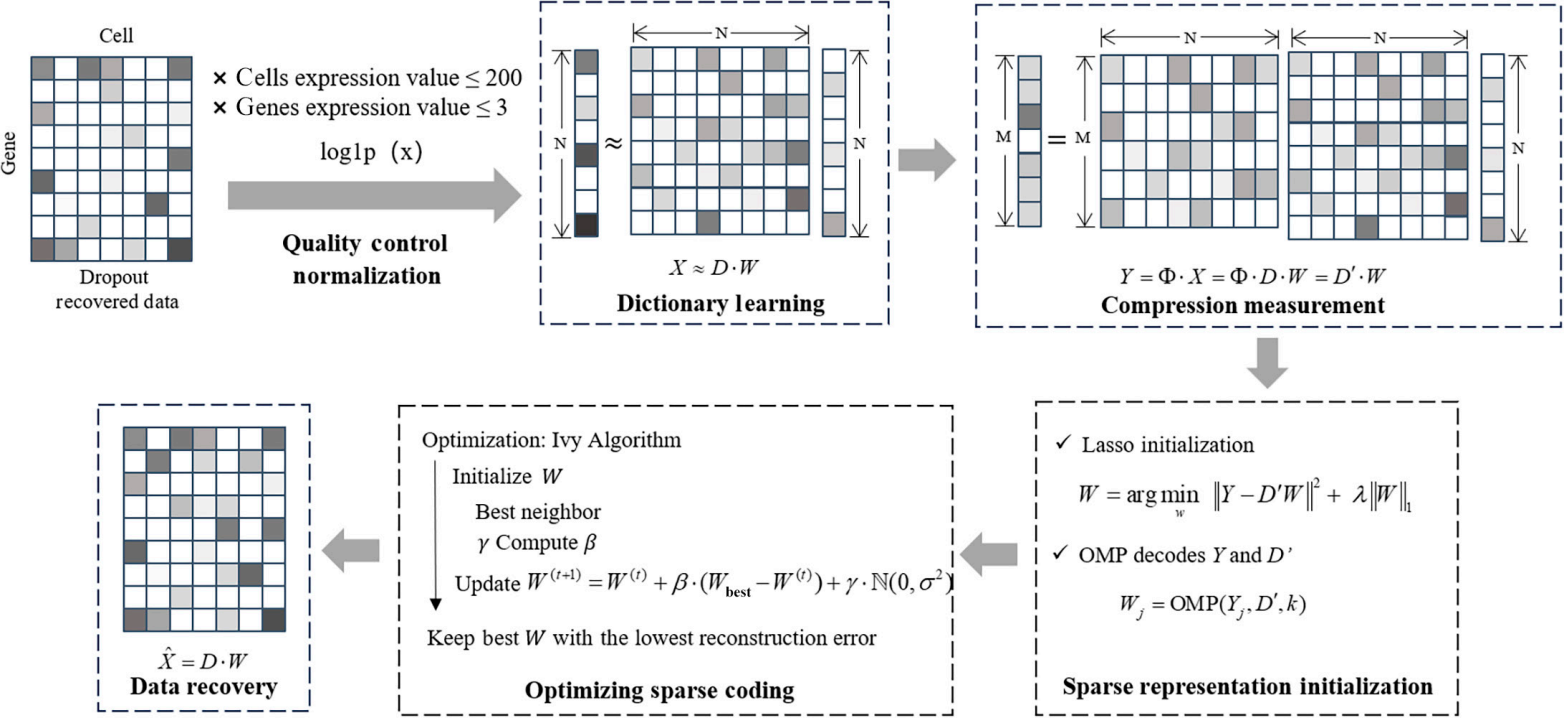
Compressed-sensing-driven optimization reconstruction module.

To further improve the structural preservation and denoising compression of the preliminarily recovered data, we introduce the CSO module. This module takes the preliminary recovery matrix generated by the SGM as input and sequentially performs sparse dictionary construction, compressed measurement generation, sparse coefficient decoding, Ivy-based optimization search, and final reconstruction, forming a closed-loop refinement process. The overall workflow is illustrated in Fig. 4.

(a) Sparse dictionary learning

To uncover the underlying low-dimensional structures within the scRNA-seq data, we first perform sparse dictionary decomposition on the recovered gene expression matrix $\hat{X}$ from the previous stage, approximating it as the product of a dictionary matrix $D\in {\mathbb{R}}^{g\times d}$ and a sparse coefficient matrix $W\in {\mathbb{R}}^{d\times n}$:

$$\hat{X}\approx D\cdot W,$$ (20)

where *g* denotes the number of genes, *n* represents the number of cells, and d≪g indicates the dimensionality of the sparse encoding. To obtain a well-structured dictionary *D*, we apply singular value decomposition to decompose $\hat{X}$ as

$$\hat{X}\approx U\cdot S\cdot V^{T},$$ (21)

The top *d* left singular vectors are selected to construct the dictionary $D=U_{\left(:,1:d\right)}$, which is then fixed and no longer updated. Subsequently, the sparse coefficient matrix *W* is solved as

$$\min_{W}{\left\|\hat{X}-D\cdot W\right\|}_{F}^{2}+\lambda_{1}{\left\|W\right\|}_{1},$$ (22)

where ${\left\|\cdot \right\|}_{F}$ denotes the Frobenius norm and $\lambda_{1}>0$ is the regularization coefficient that controls the sparsity level.

(b) Compression measurement

To reduce reconstruction complexity and enable information compression, a compressed sensing measurement matrix $\Phi \in {\mathbb{R}}^{m\times g}$ is constructed to perform linear measurements on the expression data:

$$Y=\Phi \cdot \hat{X}=\Phi \cdot D\cdot W=D^{\prime }\cdot W,$$ (23)

where m<g denotes the compressed dimension, $Y\in {\mathbb{R}}^{m\times n}$ represents the measurement matrix, and $D^{\prime }=\Phi \cdot D\in {\mathbb{R}}^{m\times d}$ refers to the sparse dictionary after projection.

(c) Sparse representation and initialization

To obtain the initial sparse coefficient matrix, this study first applies LASSO regression over the observed matrix and the sparse dictionary $D^{\prime }$ to perform global sparse encoding. The objective is to minimize the weighted reconstruction error combined with an $L_{1}$-regularization term.

$$\begin{array}{c} W=\arg\;\min_{W}{\left\|Y-D^{\prime }\cdot W\right\|}_{F}^{2}+\lambda_{2}{\left\|W\right\|}_{1}, \end{array}$$ (24)

where $\lambda_{2}$ controls the overall sparsity level of the resulting coefficient matrix.

For each column of the observation matrix $Y_{j}\in {\mathbb{R}}^{m}$, OMP is employed for sparse decoding, ensuring that the number of non-zero elements in each column is fixed to the predefined sparsity level *k*. This enhances the consistency and controllability of the sparse representation. The corresponding sparse representation is estimated as

$$W_{j}^{\left(0\right)}=\mathrm{OMP}\left(Y_{j},\;D^{\prime },\;k\right),$$ (25)

where *k* denotes the sparsity parameter controlling the number of non-zero elements. In this study, the LASSO- and OMP-derived coefficients are used as structured initial solutions for the subsequent Ivy-based refinement, rather than being replaced by the population-based search procedure. This design is consistent with the role of Ivy described in the “Nature-inspired optimization algorithms” section, namely, as a task-specific optimization strategy integrated into the reconstruction framework.

(d) Ivy Algorithm optimization

After initialization, the Ivy Algorithm is introduced to refine the sparse coefficient matrix W under the reconstruction objective defined for the CSO module. Each column of W is treated as an individual in the population, and the population evolves iteratively according to the Ivy update mechanism. In the *t*-th iteration, the position of the *i*-th individual is updated as follows:

$$W_{i}^{\left(t+1\right)}=W_{i}^{\left(t\right)}+\beta \cdot \left(W_{\text{best}}-W_{i}^{\left(t\right)}\right)+\gamma \cdot N\left(0,\;\sigma^{2}\right),$$ (26)

where $W_{t}^{\left(i\right)}$ denotes the *i*-th individual, i.e., the current solution; $W_{\text{best}}$ represents the current best-performing individual in terms of fitness. β is the growth factor, which controls the growth magnitude toward the optimal neighbor, while γ is the random expansion factor that introduces Gaussian noise $N\left(0,\;\sigma^{2}\right)$ to enhance exploration diversity.

The growth factor β adopts a dynamic adjustment mechanism, which takes into account the Euclidean distance $r_{\mathrm{ij}}$ between the current individual and the current best solution:

$$\begin{array}{c} \beta =\beta_{0}\cdot \exp\left(-\mu \cdot r_{\mathrm{ij}}\right),r_{\mathrm{ij}}={\left\|W_{i}-W_{j}\right\|}_{F}, \end{array}$$ (27)

where *β*_0_ denotes the initial growth rate, μ is the decay factor that controls the convergence speed of the growth factor, and $r_{\mathrm{ij}}$ quantifies the structural difference between the individual and the global optimum.

In contrast to the generic Ivy fitness introduced in Eq. (10), the optimization target in the CSO module is defined by the task-specific reconstruction criterion. The fitness function is defined as the Pearson-correlation-based reconstruction criterion for each column:

$$F\left(W_{j}\right)=1-\mathrm{PCC}\left({\hat{X}}_{:,j},\;D^{\prime }\cdot W_{j}\right),$$ (28)

where $\mathrm{PCC}\left(\cdot \right)$ denotes the PCC between the reconstructed output ${\mathrm{DW}}_{j}$ and the recovered expression matrix ${\hat{X}}_{:,j}$. Under this task-specific definition, a higher correlation indicates a lower reconstruction error and thus a better individual fitness. Accordingly, Eq. (28) should be understood as the reconstruction-specific instantiation of the general Ivy fitness mechanism described in the “Nature-inspired optimization algorithms” section, rather than a repetition of Eq. (10). The iterative optimization terminates when either the improvement in reconstruction error over 2 consecutive iterations falls below a predefined threshold ε or the maximum number of iterations $T_{\max}$ is reached.

(e) Data recovery

The final sparse coefficient matrix $W^{*}$ is obtained through the aforementioned optimization process, and the reconstructed gene expression matrix is given by

$$\tilde{X}=D\cdot W^{*},$$ (29)

where $\tilde{X}\in {\mathbb{R}}^{g\times n}$ denotes the gene expression matrix reconstructed via sparse optimization. This process completes the whole pipeline from initial recovery to compression and dimension reduction, followed by sparse reconstruction, demonstrating strong performance in preserving structural consistency and denoising capability.
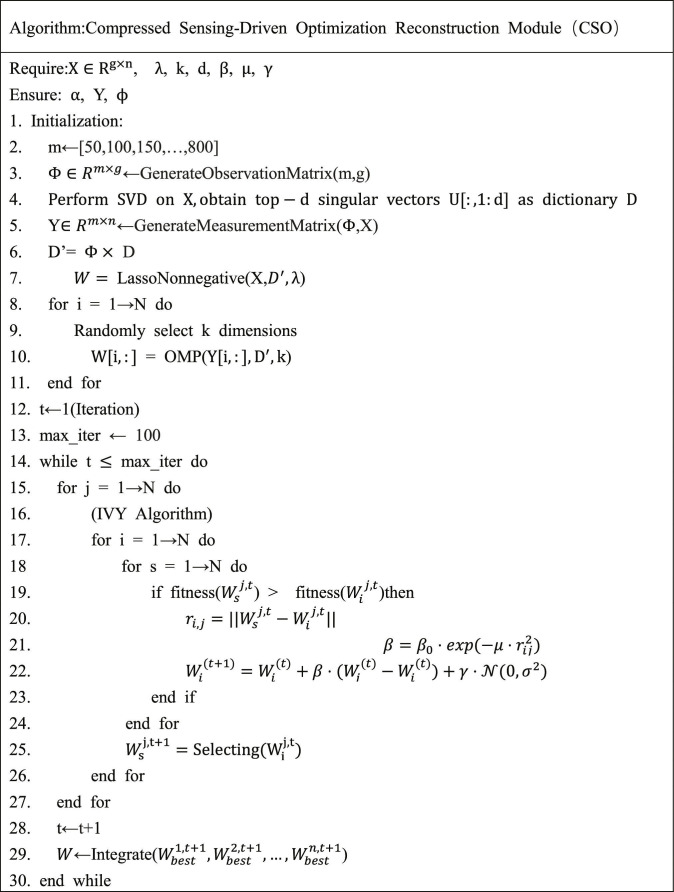


### Evaluation metrics

To comprehensively evaluate the performance of different methods in the scRNA-seq data recovery task, this study adopts multiple evaluation metrics, including MSE, MAE, PCC, and COS. These metrics are used to quantitatively analyze both the overall recovery performance and local reconstruction details. For mask-augmented experiments, these metrics are computed against known reference values. For real unmasked datasets, they are interpreted together with downstream analyses to provide a more complete evaluation of recovery performance. To avoid confusion with compressed sensing, cosine similarity is abbreviated as COS throughout this paper. The specific definitions are as follows:

• MSE:

$$\mathrm{MSE}=\frac{1}{N}\sum_{i=1}^{N}{\left(x_{i}-{\hat{x}}_{i}\right)}^{2}$$ (30)

where $x_{i}$ and ${\hat{x}}_{i}$ denote the original and recovered values of the *i*-th element, respectively, and *n* represents the total number of samples. MSE measures the average squared reconstruction error between the recovered results and the reference values, providing an intuitive reflection of the overall error magnitude.

• MAE:

$$\mathrm{MAE}=\frac{1}{N}\sum_{i=1}^{N}\left|x_{i}-{\hat{x}}_{i}\right|$$ (31)

MAE measures the mean absolute deviation between the recovered results and the reference values, providing an intuitive reflection of the overall error magnitude.

• PCC:

$$\mathrm{PCC}=\frac{\sum_{i=1}^{n}\left(x_{i}-\bar{x}\right)\left({\hat{x}}_{i}-\bar{\hat{x}}\right)}{\sqrt{\sum_{i=1}^{n}{\left(x_{i}-\bar{x}\right)}^{2}\sqrt{\sum_{i=1}^{n}{\left({\hat{x}}_{i}-\bar{\hat{x}}\right)}^{2}}}}$$ (32)

where $\bar{x}$ and $\bar{\hat{x}}$ denote the mean values of $x_{i}$ and ${\hat{x}}_{i}$, respectively. PCC is used to evaluate the correlation between the recovered data and the real data in terms of their variation trends.

• COS:

$$\mathrm{COS}=\frac{\sum_{i=1}^{n}x_{i}{\hat{x}}_{i}}{\sqrt{\sum_{i=1}^{n}{x_{i}}^{2}\sqrt{\sum_{i=1}^{n}{{\hat{x}}_{i}}^{2}}}}$$ (33)

COS reflects the similarity in vector direction between the recovered data and the real data; the closer the value is to 1, the higher the consistency between them. In this study, COS is used as an auxiliary indicator of structural consistency in expression patterns.

To more precisely evaluate recovery behavior across different parts of the data, the entries are partitioned into 2 categories: the zero-entry region and the non-zero region:

The zero-entry region refers to positions observed as zeros in the adopted input representation. Because the signal magnitude in this region is often close to zero, correlation-based metrics such as PCC and COS may become unstable under small numerical fluctuations. Therefore, only MSE and MAE are used for quantitative evaluation in this region. For clarity in subsequent metric reporting, the evaluation metrics for this region are denoted as Z_MSE and Z_MAE. For mask-augmented experiments, this region includes artificially masked entries with known reference values; for real unmasked datasets, these metrics are reported as descriptive statistics on observed-zero positions.

The non-zero region refers to positions in the original data with non-zero values, representing observed expression signals. To comprehensively assess recovery performance in this region, 4 metrics are calculated, MSE, MAE, PCC, and COS, which together evaluate both the numerical accuracy and expression consistency of the recovered data. Correspondingly, the evaluation metrics in this region are denoted as NZ_MSE, NZ_MAE, NZ_PCC, and NZ_COS.

In the subsequent experiments, performance evaluation is conducted based on this metric system. Specifically, mask-augmented experiments provide reference-based reconstruction evidence, whereas analyses on real datasets are further complemented by downstream evaluations such as clustering structure and DE consistency. This design provides a more comprehensive assessment of recovery performance across different evaluation settings.

## Experiment

### Data sources

To systematically evaluate the recovery performance of the proposed method, we selected 3 publicly available single-cell transcriptomic datasets of breast cancer: GSE123358, GSE124989, and GSE147326.

Before model construction and analysis, all raw expression matrices were standardized using a unified preprocessing pipeline. Specifically, following conventional quality control criteria for single-cell data, low-quality cells with fewer than 200 detected genes were excluded, as well as low-complexity genes expressed in fewer than 3 cells. This step effectively removes sequencing noise, dead or dying cells, and low-expression pseudogenes, thereby enhancing the robustness and reliability of downstream analyses.

**Fig. 5.**
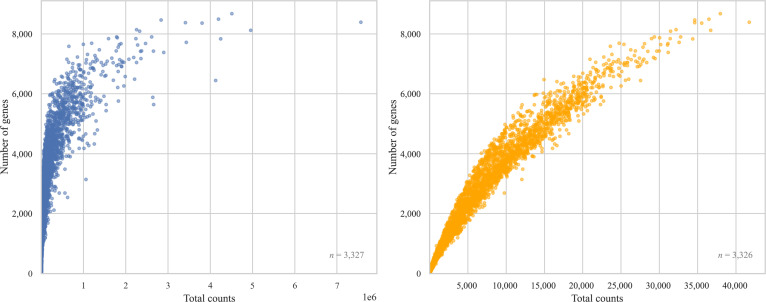
Comparison chart of cell expression quality before and after preprocessing of the GSE147326 dataset.

Subsequently, log normalization was applied to the retained expression matrices to compress the dynamic range of expression values. This step reduces the influence of sequencing depth variability and extreme values on model training, thereby enhancing the comparability of expression levels across cells and improving the stability of expression structures. Table 1 summarizes the dimensional information of each dataset before and after quality control. Figure 5 illustrates the changes in the relationship between total counts and the number of detected genes in the GSE147326 dataset before and after preprocessing. After quality control and normalization, the data distribution becomes more compact, the number of extreme outliers is reduced, and the overall data quality is improved, providing a more reliable basis for subsequent recovery analysis.

**Table 1.** Dataset introduction before and after quality control

Dataset	Library/protocol	Sequencer model	Before	After
GSE123358	Plate-based SMARTer	Illumina HiSeq 2000	21 × 48,162	21 × 17,554
GSE124989	Smart-seq2	Illumina NextSeq 500	121 × 59,838	121 × 18,243
GSE147326	ICELL8 Single-Cell System	Illumina NovaSeq 6000	3,510 × 16,521	3,327 × 16,393

### Parameter settings

To systematically evaluate the stability and robustness of the proposed integrated recovery framework, GSCI, under different hyperparameter settings, we conducted parameter sensitivity analyses on the GSE124989 dataset. These experiments covered key hyperparameters from both major modules of the framework. Detailed experimental settings are provided in the “Sensitivity analysis” section.

The key hyperparameters involved in the GSCI framework can be categorized into 2 groups: (a) training-related parameters in the SGM, such as learning rate, batch size, and latent dimension, and (b) optimization-related parameters in the CSO, including growth factor, decay rate, and population size. The final adopted values and descriptions for each hyperparameter are summarized in Table 2. Unless otherwise specified, these values were fixed in the main experiments.

**Table 2.** Parameter settings used in the experiment

Number	Parameter	Value	Description
1	*dim*	32	Dimensions of latent-space variables
2	$\alpha_{\mathrm{VAE}}$	0.1	Refactoring loss weights
3	$\beta_{\mathrm{GAN}}$	0.1	Generate loss weights
4	${\mathrm{lr}}_{G}$	$1\times 10^{-4}$	Generator and encoder learning rate
5	${\mathrm{lr}}_{D}$	$5\times 10^{-5}$	Discriminator learning rate
6	*batch_size*	128	Batch size
7	*epoch*	200	Maximum number of training epochs for SGM
8	*s*	10	Sparsity
9	*m*	400	Observations during the compressed-sensing phase
10	*N*	40	Population size
11	*max_iter*	100	Maximum number of iterations for CSO
12	β	1.0	Growth factor
13	γ	0.5	Random expansion factor

CSO, compressed-sensing-driven optimization reconstruction module; SGM, structure-guided generative module

In addition, to ensure the fairness of comparative evaluations, all baseline methods (including MAGIC, DCA, scImpute, etc.) were implemented strictly following their default hyperparameter settings as reported in the original papers or official repositories, without any additional tuning. No extra dataset-specific adjustment was introduced for the baseline methods in the main comparisons. This setting facilitates an objective assessment of the proposed method’s performance under consistent experimental conditions.

### Ablation experiment

To evaluate the individual contributions and synergistic effects of each component in the GSCI framework on expression recovery and structural preservation, we conducted ablation studies on 3 breast cancer scRNA-seq datasets (GSE123358, GSE124989, and GSE147326). Eight model variants were designed by selectively removing or retaining specific functional modules, and their results were compared with those of the whole model. The corresponding results are presented in Tables 3 to 5.

**Table 3.** Ablation study results on the GSE123358 dataset. Bold text marks the best performance value for each metric among all ablation groups.

Method	MSE	MAE	PCC	COS	Z_MSE	Z_MAE	NZ_MSE	NZ_MAE	NZ_PCC	NZ_COS
w/o Ivy	2.7656	0.9500	0.8818	0.9227	**0.5923**	**0.2904**	5.1332	1.6685	0.8036	0.9324
w/o Sparsity	7.3308	2.0691	0.5891	0.7776	5.3851	1.6087	9.4537	2.5706	0.4514	0.8971
w/o Dictionary	3.8168	1.1764	0.8206	0.8914	1.7380	0.5596	6.0815	1.8484	0.7275	0.9216
CSO Only	2.9892	1.0022	0.8703	0.9160	0.9255	0.3611	5.2375	1.7007	0.7910	0.9300
SGM Only	2.0824	0.9429	0.9057	0.9421	2.6282	1.0113	1.4878	0.8683	0.9015	0.9838
w/o Generator	6.6643	1.9521	0.6353	0.8006	4.6059	1.5860	8.9067	2.3509	0.4170	0.8892
w/o Encoder	8.0503	2.0390	0.6069	0.7853	2.0058	1.0115	14.6354	0.3418	0.3418	0.8675
Ours	**0.6561**	**0.4098**	**0.9702**	**0.9821**	0.9320	0.3590	**0.4650**	**0.3556**	**0.9768**	**0.9958**

COS, cosine similarity; MAE, mean absolute error; MSE, mean squared error; PCC, Pearson correlation coefficient

**Table 4.** Ablation study results on the GSE124989 dataset. Bold text marks the best performance value for each metric among all ablation groups.

Method	MSE	MAE	PCC	COS	Z_MSE	Z_MAE	NZ_MSE	NZ_MAE	NZ_PCC	NZ_COS
w/o Ivy	1.6411	0.7873	0.8086	0.8614	1.3907	0.6195	2.1942	1.1580	0.7822	0.9480
w/o Sparsity	4.1254	1.5751	0.3622	0.5927	2.5253	1.2158	7.6593	2.3685	0.1849	0.8810
w/o Dictionary	2.1131	0.9118	0.7432	0.8165	1.5474	0.6696	3.3624	1.4469	0.6823	0.9198
CSO Only	1.3876	0.6914	0.8377	0.8816	0.7609	0.4336	1.2608	0.9305	0.7479	0.9305
SGM Only	1.5321	0.8324	0.8284	0.8715	1.5362	0.7795	1.5455	0.9561	0.8228	0.9670
w/o Generator	2.7209	1.2129	0.6660	0.7601	2.2666	1.0572	3.7243	1.5568	0.5728	0.9218
w/o Encoder	2.8951	1.2131	0.6165	0.7330	1.8415	0.9314	5.2219	1.8352	0.4718	0.8928
Ours	**0.6085**	**0.4808**	**0.9361**	**0.9514**	**0.3618**	**0.3253**	**1.1414**	**0.8242**	**0.8718**	**0.9765**

**Table 5.** Ablation study results on the GSE147326 dataset. Bold text marks the best performance value for each metric among all ablation groups.

Method	MSE	MAE	PCC	COS	Z_MSE	Z_MAE	NZ_MSE	NZ_MAE	NZ_PCC	NZ_COS
w/o Ivy	0.4851	0.4080	0.8071	0.8307	0.3151	0.3104	1.3575	0.9089	0.7564	0.9269
w/o Sparsity	1.0362	0.6344	0.4865	0.5738	0.4897	0.4533	3.8397	1.5635	0.4223	0.8488
w/o Dictionary	0.5163	0.3757	0.7872	0.8160	0.2756	0.2469	1.7513	1.0364	0.7195	0.9035
CSO Only	0.4217	0.3788	0.8275	0.8504	0.2598	0.1892	1.6146	0.9897	0.7769	0.9101
SGM Only	0.4653	0.3905	0.8180	0.8385	0.3099	0.2968	1.2624	0.8712	0.7680	0.9321
w/o Generator	0.4758	0.3912	0.8133	0.8347	0.3146	0.2945	1.3030	0.8868	0.7602	0.9298
w/o Encoder	0.4694	0.3831	0.8149	0.8371	0.3081	0.2853	1.2966	0.8847	0.7632	0.9300
Ours	**0.3703**	**0.3429**	**0.8537**	**0.8694**	**0.2405**	**0.1979**	**1.2547**	**0.8683**	**0.7700**	**0.9328**

Note: To investigate the contribution of each component within the generative and sparse compressed sensing imputation (GSCI) framework to the overall performance, we design the following model variants for the ablation study: • w/o Ivy: removes the Ivy optimization strategy from the CSO module, retaining only the compressed-sensing-based sparse reconstruction framework. This variant is used to assess the role of global search strategies in modeling expression consistency. • w/o Sparsity: eliminates the $L_{1}$sparsity regularization term from the CSO module, keeping only least squares reconstruction, to evaluate the contribution of sparsity constraints in error control. • w/o Dictionary: removes the dictionary learning part of the CSO module and directly encodes using the raw expression matrix, aiming to verify the role of learned expression bases in structural recovery. • CSO Only: completely discards generative modeling and retains only the CSO reconstruction module to examine the standalone recovery ability of the compressed sensing framework without prior generative support. • SGM Only: retains the generative model with a combined variational autoencoder (VAE) and generative adversarial network (GAN) architecture but removes the CSO reconstruction stage, relying solely on generative modeling for expression recovery. • w/o Generator: removes the discriminator, keeping only the VAE component of the SGM to analyze the reconstruction capacity of the encoder–decoder architecture without adversarial training. • w/o Encoder: removes the encoder and uses only the adversarial generative network to explore the recovery ability of adversarial structures without latent-space learning. • Ours (GSCI): the complete generative compressed sensing integration model proposed in this work, combining the SGM and CSO modules for joint modeling.

The ablation results show that the complete GSCI model maintains a consistently favorable performance profile across the evaluated metrics. It not only yields the lowest reconstruction errors (MSE and MAE) but also reaches the highest levels in correlation metrics (PCC and COS) and structure-related indicators in the non-zero entries (NZ_PCC and NZ_COS). These findings suggest that GSCI better preserves expression consistency and non-zero structural information under the current evaluation setting. In contrast, the w/o Ivy variant consistently shows reduced similarity in PCC and COS. Additionally, its structure-preserving performance, as reflected by NZ_COS and NZ_PCC, is markedly diminished, indicating that the Ivy-guided refinement contributes to stable structural modeling.

Furthermore, the w/o Sparsity variant exhibits volatile performance, with substantially increased MSE values across multiple datasets and PCC scores falling below 0.4. The structure-related indicators in non-zero-entry also deteriorate substantially, supporting the role of the sparsity constraint in controlling reconstruction complexity and preserving key expression structure. In comparison, the w/o Dictionary variant shows relatively controlled reconstruction errors; however, the structural preservation metrics—particularly NZ_COS—deteriorate markedly. This indicates that directly encoding the raw expression matrix constrains the compression representation’s ability to adapt to local expression patterns.

On the other hand, the CSO Only variant achieves relatively reasonable expression reconstruction performance, but still underperforms compared to the Ours model across all primary metrics. This suggests that the generative module provides useful prior support for modeling expression distributions. In contrast, although the SGM Only variant performs moderately well in specific metrics, such as the PCC, it fails to match the complete GSCI model in terms of reconstruction fidelity and structural consistency, particularly in the non-zero entries. Additionally, the w/o Generator and w/o Encoder variants, which rely on incomplete generative modeling, exhibit considerable variability across multiple metrics. Their markedly lower NZ_PCC and NZ_COS scores further indicate a limited ability to capture the structural patterns underlying dropout stably.

In summary, the ablation study systematically reveals the synergistic and complementary roles of each submodule within the GSCI framework. The generative components substantially enhance reconstruction performance in the early recovery stage, while the sparsity optimization and dictionary learning modules reinforce structural constraints in expression representation. The CSO module further improves coefficient refinement and reconstruction consistency under the adopted objective. Compared to single-module or simplified variants, GSCI consistently outperforms the ablated versions in multiple evaluation metrics, highlighting the synergistic effect of the SGM and CSO modules in single-cell expression recovery.

### Results of different methods

To comprehensively evaluate the proposed framework, we compared it with representative algorithms covering graph diffusion, deep variational/autoencoding, statistical/matrix factorization, and adversarial generators on the 3 breast cancer datasets (GSE123358, GSE124989, and GSE147326). The panel included MAGIC, DCA, SAVER, scVI, ALRA, scImpute, scIGANs, scMultiGAN, and PCA/ICA/NMF. All tools were run from the authors’ official releases with the same preprocessing protocol as permitted by each method: MAGIC (Python magic-impute), DCA (Python dca), SAVER (R SAVER), scVI (Python scvi-tools), ALRA (R ALRA), scImpute (R scImpute), and PCA/ICA/NMF (scikit-learn). For the 2 adversarial generators, we used the official Python implementations without architectural changes; scIGANs was trained on raw counts with library-size normalization as specified in its documentation, and scMultiGAN followed its log1p-normalized input convention with the default multi-generator/discriminator setting. When a method required a particular input domain (e.g., raw counts for SAVER/scImpute), we honored that requirement; otherwise, methods were applied to the same preprocessed matrices used by our approach to minimize confounding due to normalization differences.

**Fig. 6.**
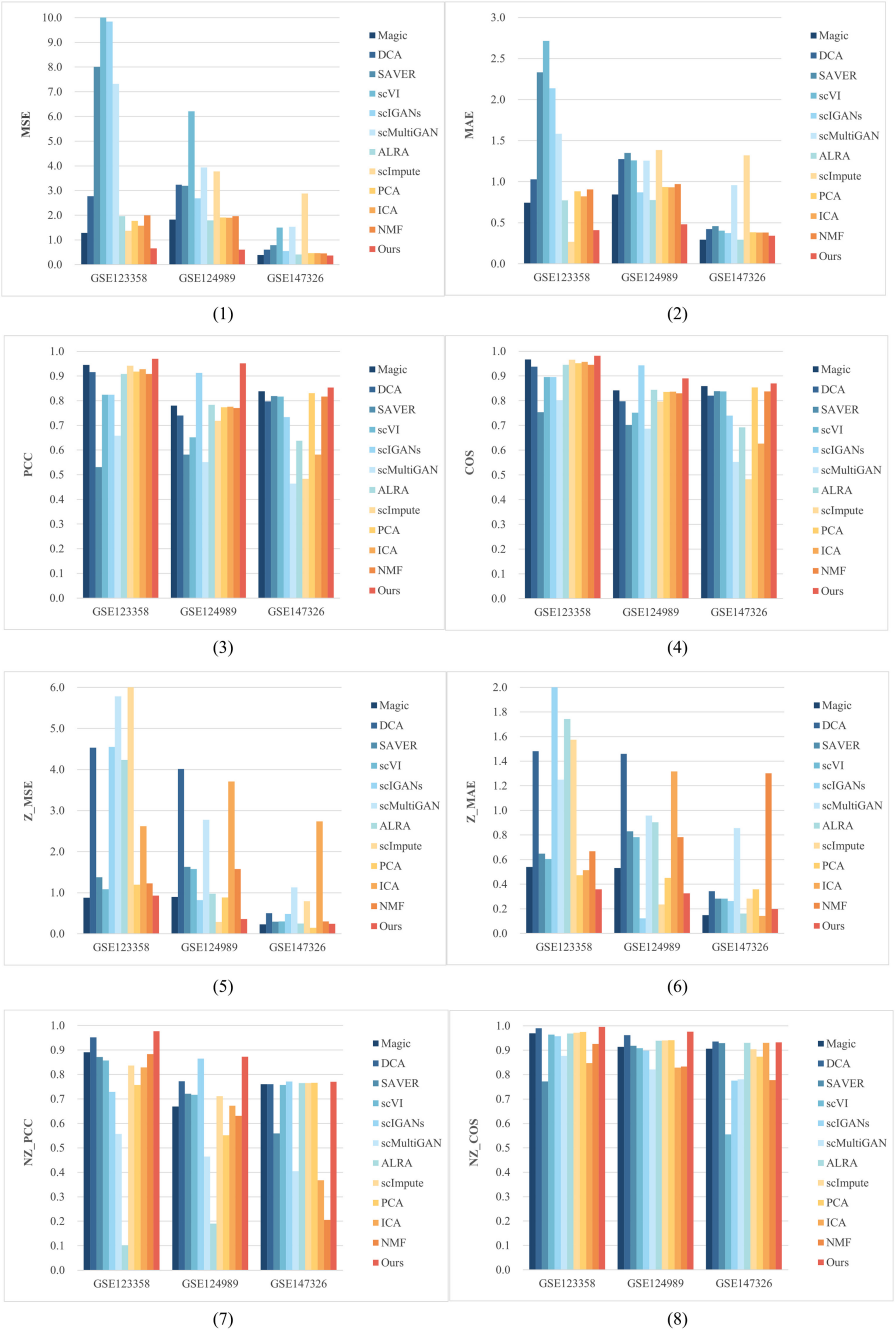
Comparison of 8 evaluation metrics across 3 single-cell datasets (GSE123358, GSE124989, and GSE147326) for different recovery methods.

Across the 8 metrics reported in Fig. 6 (global MSE/MAE/PCC/COS; zero-entry Z_MSE/Z_MAE; non-zero-entry NZ_PCC/NZ_COS), the proposed method shows consistently strong performance on all 3 datasets. In the global view, its bars are generally among the lowest for error and among the highest for correlation/similarity, indicating accurate reconstruction while maintaining overall structure. On zero-entry positions, the descriptive Z_MSE and Z_MAE values remain relatively low under the current evaluation setting. Among non-zero entries, the structural metrics remain high, indicating better preservation of observed expression patterns.

At the same time, several complementary patterns are evident across the baseline methods. Graph diffusion (MAGIC) can improve global correlation but tends to produce smoother manifolds, which is accompanied by weaker non-zero structural metrics on larger cohorts. Variational autoencoders (DCA and scVI) are competitive on global similarity and approach our method in some panels; however, their zero-entry error statistics are often higher, indicating less conservative behavior on observed-zero positions. Statistical and matrix-factorization methods (ALRA, SAVER, scImpute, and PCA/ICA/NMF) remain efficient and are occasionally competitive on small datasets, but they tend to underfit nonlinear manifolds and zero-inflated structures, leading to lower non-zero structural scores. The adversarial generators (scIGANs and scMultiGAN) capture manifold structure relatively well and are close on some global similarities; however, they show greater variability across datasets, suggesting higher sensitivity to dataset characteristics and dropout severity.

### Sensitivity analysis

**Fig. 7.**
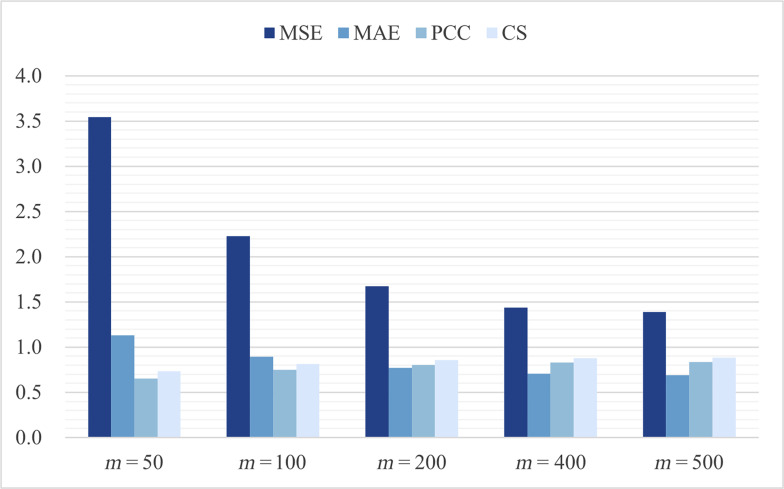
Comparison of recovery performance indicators under different measurement numbers (*m*). As the number of measurements increases, the indicators continue to improve, indicating that sufficient compressed measurements play a crucial role in enhancing accuracy.

**Fig. 8.**
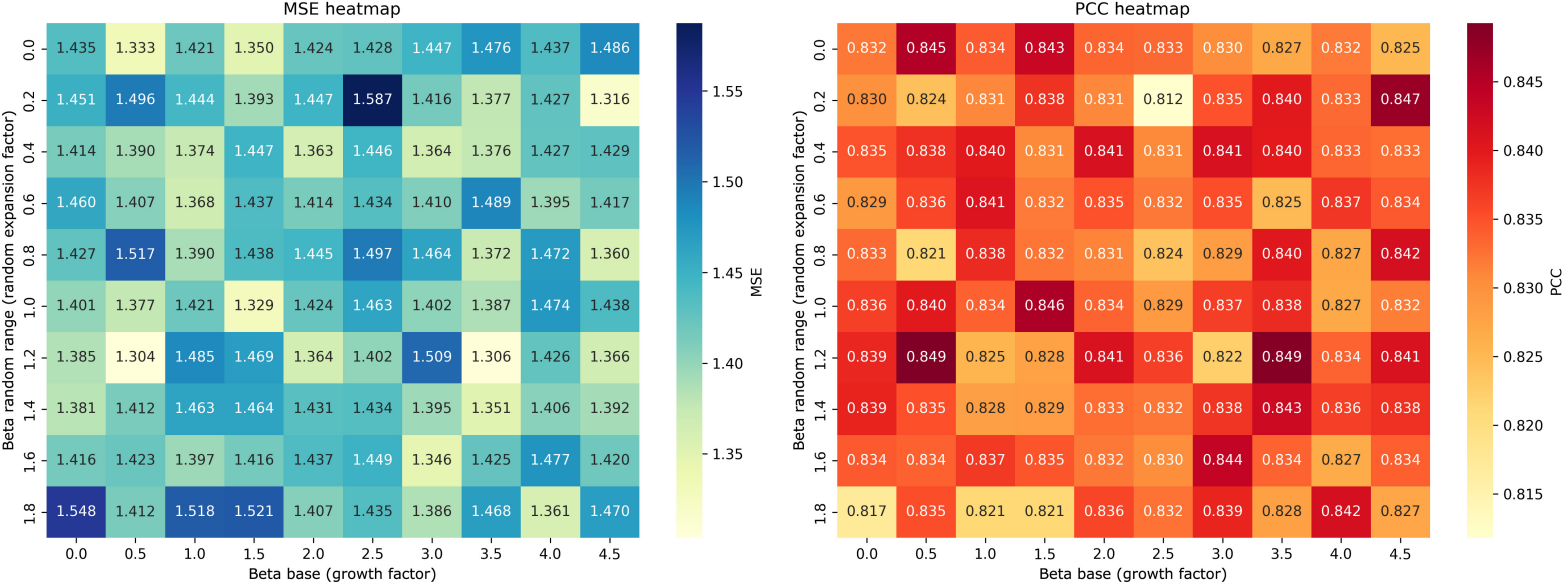
Heatmap analysis of key parameters in the CSO module on model performance.

To evaluate the robustness of GSCI to hyperparameter variation, we conducted sensitivity analyses on the GSE124989 dataset. The examined parameters included the number of compressed measurements (*m*), latent dimension (*dim*), reconstruction loss weight ($\alpha_{\mathrm{VAE}}$), generative loss weight ($\beta_{\mathrm{GAN}}$), population size (*N*), and the Ivy-related parameters β and γ. The corresponding results are summarized in Table 6, Fig. 7, and Fig. 8.

**Table 6.** Recovery performance indicators under different hyperparameter settings. Bold numbers mark the optimal performance for each metric under different hyperparameter settings.

Type		MSE	MAE	PCC	COS	Z_MSE	Z_MAE	NZ_PCC	NZ_COS
dim	16	1.2668	0.7406	0.8586	0.8940	1.3124	0.6981	0.8698	0.9760
	**32**	**1.2625**	**0.7402**	**0.8589**	**0.8942**	**1.3055**	**0.6967**	**0.8709**	**0.9762**
	64	1.2799	0.7432	0.8578	0.8934	1.3446	0.7081	0.8699	0.9761
	128	1.2751	0.7433	0.8577	0.8933	1.3264	0.7024	0.8700	0.9761
	256	1.2871	0.7480	0.8565	0.8924	1.3416	0.7082	0.8687	0.9760
$\alpha_{\mathrm{VAE}}$	**0.1**	**1.2518**	**0.7355**	0.8598	0.895	**1.2898**	**0.6898**	**0.8717**	**0.9763**
	0.3	1.2680	0.7403	0.8587	0.8940	1.3233	0.7013	0.8714	0.9763
	0.5	1.2652	0.7385	0.8591	0.8944	1.3211	0.6997	0.8710	0.9763
	0.7	1.2746	0.7414	**0.8583**	**0.8937**	1.3341	0.7044	0.8699	0.9760
	0.9	1.2557	0.7371	0.8597	0.8948	1.2965	0.6929	0.8704	0.9761
$\beta_{\mathrm{GAN}}$	0.01	1.2698	0.7412	0.8584	0.8938	1.3229	0.7014	0.8708	0.9762
	0.05	1.2624	0.7412	0.8586	0.8941	1.2986	0.8424	0.8700	0.9676
	**0.10**	**1.2461**	**0.7318**	**0.8611**	**0.8958**	**1.2950**	**0.6909**	**0.8726**	**0.9765**
	0.15	1.2671	0.7425	0.8581	0.8938	1.3070	0.6976	0.8699	0.9760
	0.20	1.2677	0.7387	0.8591	0.8943	1.3297	0.7025	0.8713	0.9754
N	10	1.4352	0.7052	0.8322	0.8776	0.7889	0.4431	0.7407	0.9276
	20	1.4337	**0.7051**	**0.8323**	0.8777	0.7888	0.4432	0.7411	0.9278
	30	1.4356	0.7061	0.8321	0.8775	0.7906	0.4443	0.7408	0.9277
	40	1.4350	0.7060	0.8322	0.8776	0.7909	0.4443	0.7410	0.9278
	50	**1.4337**	0.7051	0.8323	**0.8377**	**0.7880**	**0.4430**	**0.7411**	**0.9278**

As shown in Fig. 7, increasing the number of compressed measurements generally reduces MSE and MAE while improving PCC and COS, indicating that a sufficient measurement budget benefits sparse reconstruction. However, the improvement becomes marginal when *m* increases from 400 to 500. Therefore, considering both reconstruction performance and computational cost, we use *m* = 400 in the subsequent experiments.

Table 6 shows that the model remains relatively stable across different values of *dim*, $\alpha_{\mathrm{VAE}}$, $\beta_{\mathrm{GAN}}$, and *N*. Among the tested settings, *dim* = 32 yields the most favorable overall trade-off between reconstruction error and structural consistency. For the loss weights, $\alpha_{\mathrm{VAE}}$ = 0.1 and $\beta_{\mathrm{GAN}}$ = 0.1 provide the best overall results under the current evaluation setting. In addition, the performance varies only slightly across different population sizes, suggesting that the framework is not highly sensitive to *N*; therefore, *N* = 40 is retained as the default configuration.

To further analyze the Ivy-related parameters, we evaluated the joint effect of the growth factor β and the stochastic expansion factor γ on a 2-dimensional grid, using MSE and PCC as the main criteria. As shown in Fig. 8, favorable performance is concentrated in a relatively broad region rather than at a single sharply tuned point. In particular, β = 0.5 and γ = 1.0 provide the best overall balance between reconstruction error and expression consistency and are therefore adopted in the final configuration.

Overall, the sensitivity results show that GSCI maintains relatively stable performance under moderate hyperparameter perturbations. These results support that the reported performance is not driven by a narrowly tuned parameter setting but reflects a reasonably robust behavior of the framework under the current experimental protocol.

### Convergence analysis

To systematically verify the numerical stability and optimization convergence capability of the proposed joint recovery framework during training, we conducted convergence experiments on the GSE124989 dataset. Specifically, we analyzed the dynamic changes during optimization within both SGM and CSO.

**Fig. 9.**
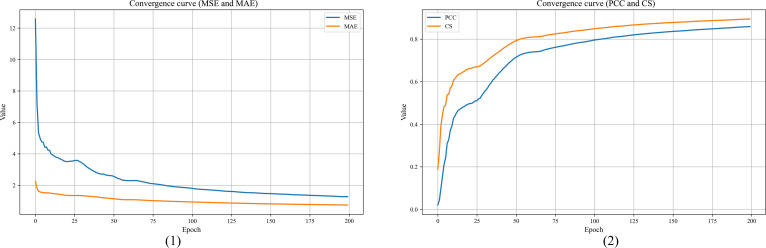
Convergence curves of global evaluation metrics during SGM training.

First, in the SGM, we monitored the evolution of performance metrics throughout the training process. Figure 9 presents the convergence curves of 4 global evaluation metrics—MSE, MAE, PCC, and COS—across consecutive training epochs. Figure 9A illustrates the downward trends of MSE and MAE, both of which decrease rapidly within the first 50 epochs, indicating that the generator quickly captures the major structural features of the expression data. Subsequently, these error metrics stabilize and converge to low-value ranges, indicating stable optimization behavior of the generative recovery process. Figure 9B illustrates the progressive increase in PCC and COS values, which rise rapidly during the early training phase and then gradually plateau, indicating a steady improvement in the similarity between generated outputs and the original expression profiles. Taken together, these trends suggest that the SGM reaches a relatively stable recovery state after a limited number of training epochs.

**Fig. 10.**
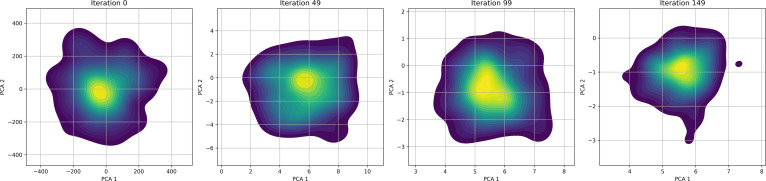
Density evolution of the sparse coefficient matrix in PCA space during CSO optimization.

Following this, we performed sparse reconstruction and expression structure refinement using the CSO framework, based on the preliminary recovery matrix generated by the SGM. To intuitively visualize the structural evolution of the sparse coefficient matrix W during optimization, we introduced density distribution plots in Fig. 10, depicting the distribution of W projected into PCA space. Initially, the sparse coefficients show a dispersed and irregular pattern, with an indistinct central structure. As iterations progress, the distribution gradually concentrates in a more compact region, and the structure becomes more transparent and more orderly. Notably, by iteration 100, the distribution pattern has largely converged, and by iteration 150, both the center and contour of the density remain almost unchanged, suggesting that the sparse coefficients evolve from an initially diffuse distribution toward a relatively stable and structured configuration.

**Fig. 11.**
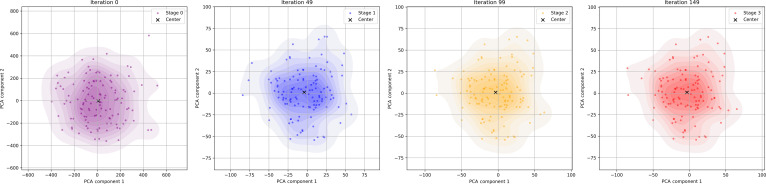
Stage-wise visualization of sparse coefficient distributions during CSO optimization.

Furthermore, Fig. 11 illustrates the distribution of the sparse coefficients at various optimization stages (iterations 0, 50, 100, and 150). From stage 0 to stage 2 (i.e., iteration 0 to 99), the sparse vectors transition from a scattered initialization to a more compact cluster centered in the coefficient space. Upon reaching stage 3 (iteration 149), the overall distribution closely resembles that of the previous stage, which is consistent with the density-based observation in Fig. 10 and further supports the conclusion that the main structural convergence is achieved around iteration 100.

In conclusion, the proposed joint recovery framework exhibits stable training dynamics and convergent optimization behavior in both the generative and sparse reconstruction phases. The results indicate that the model can reach a relatively stable state within a moderate number of training epochs and optimization iterations, supporting the feasibility of the proposed 2-stage recovery procedure for single-cell expression data.

### Performance across prescribed masking ratios

**Fig. 12.**
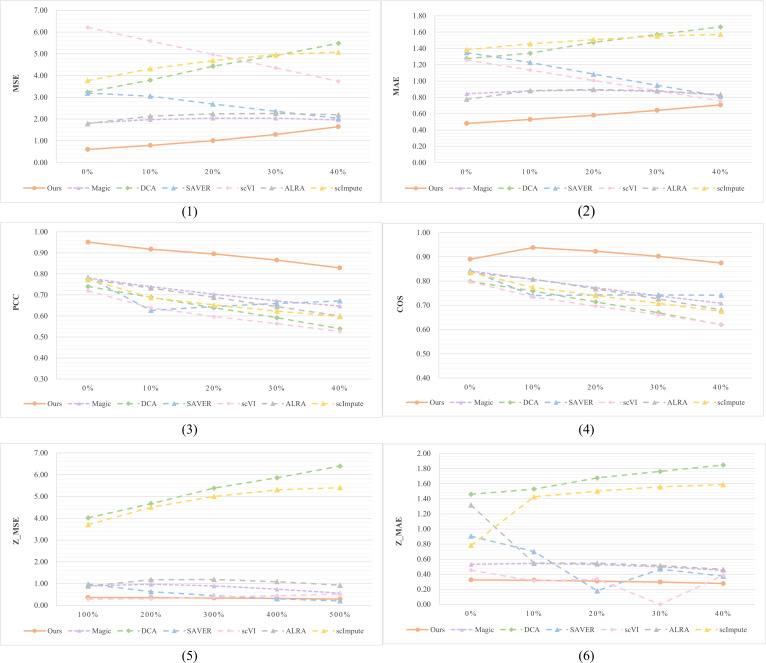
Comparison results of 6 indicators of various methods on the GSE124989 dataset under different Dropout rates.

To complement the real-data comparisons with an evaluation in which reference values are available, we construct mask-augmented datasets by stochastically hiding a prescribed proportion of non-zero entries in the original expression matrix. For each masking ratio (e.g., 0%, 10%, 20%, 30%, and 40%), the masked matrix serves as the method input while the unmodified matrix is retained as the reference, thereby largely preserving the empirical gene-cell structure and enabling controlled, reference-based benchmarking. All baselines are executed from their official releases using the input domain they require (e.g., raw counts for some statistical methods; log-normalized inputs where specified) and otherwise applied to the same preprocessed matrices as our method to minimize normalization-induced confounding. Figure 12 presents the full comparative results on GSE124989. Using the same masking protocol, we further evaluated GSCI together with representative baseline methods, including MAGIC, scVI, ALRA, and SAVER, on GSE123358 and GSE147326.

In the full comparative panel on GSE124989, all methods show the expected degradation in global error and similarity, but the patterns differ by family. Graph-diffusion approaches (e.g., MAGIC) often retain relatively high global correlation at modest masking, while exhibiting progressive oversmoothing that appears as steeper declines in COS at higher ratios. Variational/autoencoding models (e.g., DCA and scVI) are competitive on global similarity over a broad range of ratios, yet their masked-entry errors tend to rise as masking intensifies, indicating less conservative recovery behavior on masked entries. Classical statistical and matrix-factorization baselines (e.g., ALRA, SAVER, scImpute, and linear PCA/ICA/NMF when considered) remain computationally efficient and can be close on smaller matrices, but their performance generally becomes less stable as the masking ratio increases. When adversarial generators are included in the panel, they can approach leading global similarity at low–moderate masking but exhibit greater variability and higher masked-entry errors under stronger masking.

Within this landscape, the proposed framework exhibits a balanced error–structure profile across ratios: masked-entry errors (Z-MSE/Z-MAE) increase more slowly than most baselines, while global similarity (PCC/COS) remains comparatively high. In practical terms, the method limits over-filling in masked entries without unduly sacrificing overall correlation and structural consistency as masking becomes more severe. Similar trends are also observed on GSE123358 and GSE147326, indicating that the masking-based advantage of GSCI is reproducible across datasets with different sample scales and sparsity levels. These mask-augmented results provide a controlled, reference-based view of robustness across the tested masking ratios.

### Recovery of key genes

To further assess gene-level recovery behavior of various imputation methods, this study focuses on evaluating the recovery performance of key gene expressions associated with breast cancer mechanisms. We first constructed an initial list of candidate genes by integrating PubMed-curated breast cancer genes with pathogenic gene information from the KEGG disease database (H00031). This list was then intersected with the gene set used in the present study, resulting in a final selection of 22 breast-cancer-related key genes that play crucial roles in various biological processes, including DNA repair, hormone signaling pathways, the cell cycle, and signal transduction. These genes are extensively involved in the initiation and progression of breast cancer and hold substantial value in clinical diagnosis, molecular subtyping, and targeted therapy.

**Fig. 13.**
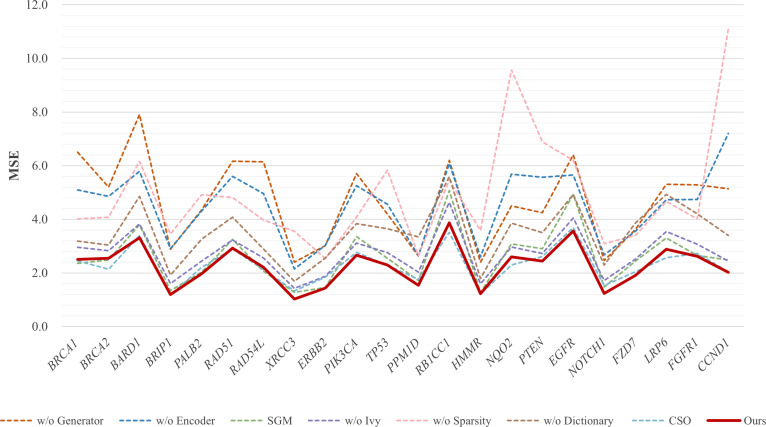
MSE change trends of 8 ablation experimental methods on 22 key genes in the GSE124989 dataset. SGM, structure-guided generative module.

**Fig. 14.**
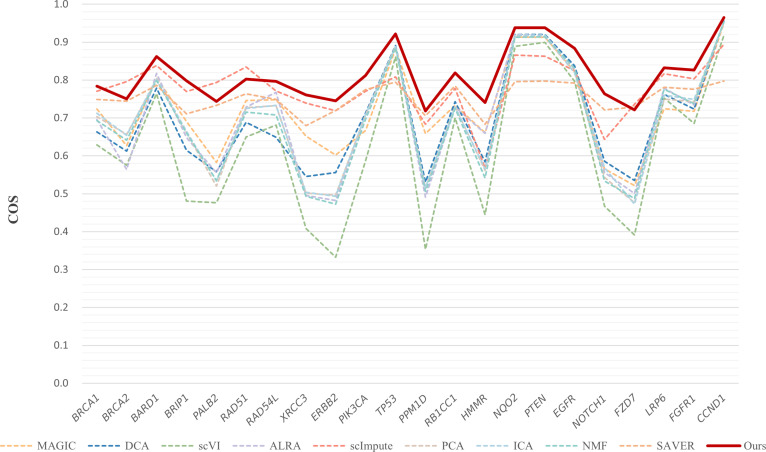
COS change trends of 22 key genes in 10 comparative experimental methods on the GSE124989 dataset.

Table 7 lists the 22 key genes used in this study along with their primary roles in breast cancer. These include genes such as *BRCA1/BRCA2* (DNA damage repair), *TP53* (tumor suppression), *ERBB2* (HER2 signaling pathway), and *ESR1/PGR* (hormone receptor pathways), all of which exhibit strong clinical and biological relevance. To systematically evaluate the recovery performance for key genes, we visualized both the recovery error and structural consistency on the GSE124989 dataset. Specifically, Fig. 13 presents the MSE trends of 8 ablation variants across 22 key genes, while Fig. 14 illustrates the COS performance of 10 comparison methods on these genes.

**Table 7.** Twenty-two key genes related to breast cancer and their functional annotations

Gene	Role in breast cancer	Optional
*BRCA1*	Germline mutations in *BRCA1* impair DNA repair and increase the risk of cancer	Tumor suppressor
*BRCA2*	*BRCA2* mutations disrupt homologous recombination, promoting tumorigenesis	Tumor suppressor
*BARD1*	*BARD1* forms a complex with *BRCA1* to facilitate DNA damage response	*BRCA1*-interacting protein
*BRIP1*	*BRIP1* participates in *BRCA1*-associated DNA repair pathways	Fanconi anemia family
*PALB2*	*PALB2* bridges *BRCA1* and *BRCA2* to support homologous recombination	Partner of *BRCA2*
*RAD51*	*RAD51* plays a central role in homologous recombination repair	Core DNA repair enzyme
*RAD54L*	*RAD54L* enhances *RAD51*-mediated strand invasion during DNA repair	HR pathway component
*XRCC3*	*XRCC3* supports DNA double-strand break repair and genomic stability	DNA repair gene
*ERBB2*	*ERBB2* is amplified in HER2-positive breast cancer and serves as a drug target	HER2 oncogene
*PIK3CA*	*PIK3CA* mutations activate the PI3K/AKT pathway, promoting tumor growth	Oncogene
*TP53*	*TP53* is a frequently mutated tumor suppressor involved in apoptosis and DNA repair	Master regulator
*PPM1D*	*PPM1D* negatively regulates p53 signaling and is amplified in some tumors	p53 pathway regulator
*RB1CC1*	*RB1CC1* modulates cell cycle progression and autophagy	Cell cycle regulator
*HMMR*	*HMMR* enhances cancer cell migration and may interact with *BRCA1*	Motility-associated gene
*NQO2*	*NQO2* regulates oxidative stress and redox balance in cancer cells	Detoxification pathway
*PTEN*	*PTEN* inhibits the PI3K/AKT pathway and is frequently deleted in cancer	Tumor suppressor
*EGFR*	*EGFR* is overexpressed in triple-negative breast cancer and guides therapy	RTK family
*NOTCH1*	*NOTCH1* is activated in TNBC and drives epithelial–mesenchymal transition	EMT signaling
*FZD7*	*FZD7* mediates WNT signaling and contributes to metastasis	WNT pathway component
*LRP6*	*LRP6* acts as a co-receptor in WNT signaling and supports proliferation	Upstream WNT regulator
*FGFR1*	*FGFR1* amplification is associated with the luminal B subtype and endocrine resistance	RTK family
*CCND1*	*CCND1* promotes G1/S transition and is frequently amplified in ER+ tumors	Cell cycle driver

HR, homologous recombination; HER2, human epidermal growth factor receptor 2; PI3K, phosphatidylinositol 3-kinase; AKT, protein kinase B (also known as Akt serine/threonine kinase); RTK, receptor tyrosine kinase; TNBC, triple-negative breast cancer; EMT, epithelial–mesenchymal transition; WNT, wingless/integrated; ER+, estrogen-receptor-positive.

As shown in Fig. 13, GSCI achieves the lowest or near-lowest MSE for the majority of key genes, showing favorable performance on clinically critical loci such as TP53, BRCA1, and PIK3CA. In addition, compared to ablation variants where any module is removed, GSCI consistently maintains lower recovery error across most genes. This observation is consistent with the ablation results at the global metric level and further supports the complementary roles of the proposed modules.

Figure 14 illustrates the comparison of COS across different recovery methods. GSCI again achieves higher structural consistency for most genes, with a relatively smooth performance curve, highlighting its ability to preserve the original structural distribution of gene expression matrices. In contrast, mainstream methods such as scVI, MAGIC, and scImpute show notable fluctuations or substantial degradation on specific genes, indicating instability in structural preservation.

By synthesizing the results from Figs. 13 and 14, it is evident that GSCI not only effectively reduces the recovery error of key genes but also maintains the structural consistency of the original expression data. This gene-level analysis complements the global metrics and downstream evaluations presented in other sections and provides additional support for the biological plausibility of the recovered data.

### External validation on heterogeneous datasets

To evaluate generalization beyond the development cohorts, we applied the complete GSCI pipeline—without dataset-specific tuning—to 6 heterogeneous scRNA-seq datasets (PBMC3k, PBMC68k, GSE102475, GSE78779, GSE48968, and GSE62270). Preprocessing followed the “Methodology” section. Table 8 summarizes the external-validation results, where for each dataset we report global error and similarity (MSE, MAE, PCC, and COS), descriptive zero-entry statistics on entries observed as zeros in the input (Z-MSE and Z-MAE), and non-zero-entry statistics (NZ-PCC, NZ-COS, NZ-MSE, and NZ-MAE).

**Table 8.** External validation across 6 heterogeneous single-cell RNA-sequencing datasets

Metrics		PBMC3k	PBMC68k	GSE102475	GSE78779	GSE48968	GSE62270
global	MSE	0.0560	0.0286	1.2757	0.1162	0.7587	0.8298
	MAE	0.1120	0.0453	0.8027	0.2367	0.5762	0.6068
	PCC	0.8944	0.9300	0.9021	0.9856	0.8943	0.9141
	COS	0.9003	0.9335	0.9380	0.9944	0.9195	0.9350
obs.-zero	Z_MSE	0.0762	0.0096	0.6853	0.2257	0.3952	0.4874
	Z_MAE	0.0168	0.0321	0.5831	0.1269	0.3645	0.4619
non-zero	NZ_PCC	0.8540	0.8519	0.7252	0.9689	0.8383	0.6805
	NZ_COS	0.9333	0.9542	0.9616	0.9964	0.9466	0.9678
	NZ_MSE	0.6569	0.9596	2.1226	0.2418	1.5584	1.8867
	NZ_MAE	0.6530	0.6943	1.1177	0.1112	0.9433	1.0540

Across these out-of-domain datasets, GSCI maintains high global similarity (PCC/COS typically around 0.89 to 0.99) with errors that scale with sparsity. PBMC3k, PBMC68k, and GSE78779 show relatively low global MSE/MAE together with strong correlation and cosine similarity, whereas GSE48968, GSE62270, and especially GSE102475 present larger zero-entry statistics, consistent with heavier sparsity and increased recovery difficulty. Importantly, expression structure among non-zero entries is largely preserved: NZ-COS remains high (≈0.94 to 0.997) across all datasets and NZ-PCC is strong on PBMC3k and GSE78779 but lower on GSE102475 and GSE62270, where increased heterogeneity and sparsity also manifest as larger NZ-MSE/NZ-MAE. These results indicate that, although reconstruction becomes more challenging in highly sparse settings, GSCI still preserves coherent expression structure among non-zero entries across heterogeneous datasets.

**Fig. 15.**
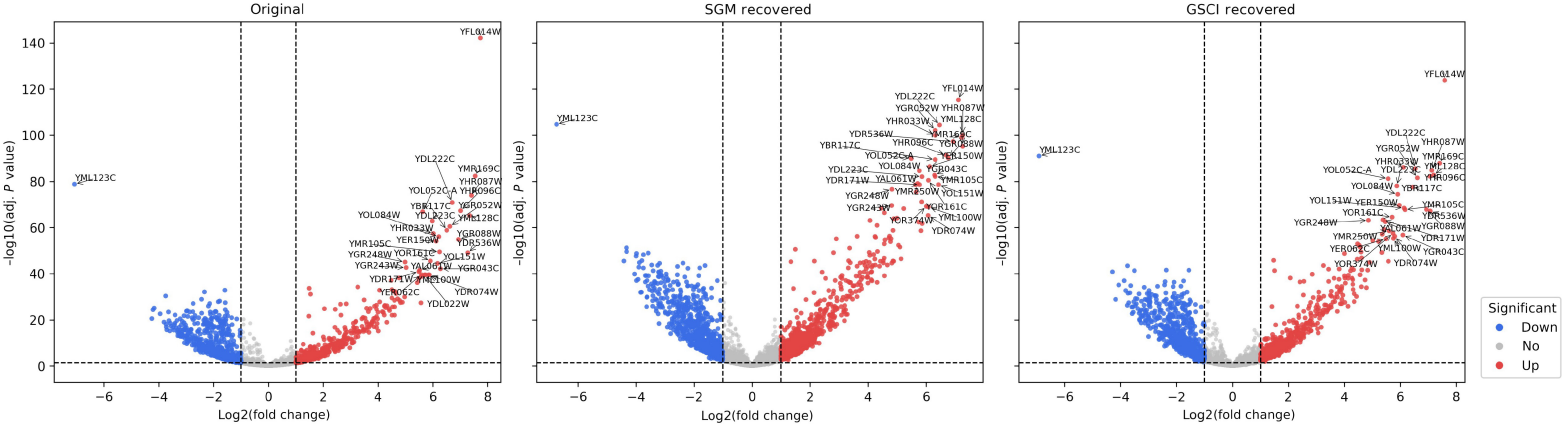
Differential expression on GSE102475 under 3 inputs.

As a downstream plausibility check on GSE102475, we performed DE analysis under identical thresholds across the original counts, an SGM-only recovery, and the final GSCI output. As visualized in Fig. 15, the volcano plots show a clearer separation of the up- and down-regulated arms after GSCI and fewer borderline calls near the decision boundaries.

## Conclusion

This study introduces GSCI, a framework that couples an SGM with a compressed-sensing-driven reconstruction module for scRNA-seq expression recovery. The framework addresses the coupled challenges of dropout-affected expression recovery and structure-preserving compact representation by combining distribution-aware preliminary recovery with sparsity-guided refinement. Across 3 breast-cancer datasets and a diverse set of comparison methods spanning graph diffusion, variational/autoencoding, statistical/matrix-factorization, linear, and adversarial approaches, GSCI maintains consistently favorable reconstruction performance and structural consistency under a unified evaluation protocol.

Beyond the in-domain comparisons, multiple complementary analyses support the robustness of the proposed framework. First, a mask-augmented evaluation with prescribed masking ratios provides a controlled, reference-based view of performance: As masking intensifies, GSCI retains higher global similarity with slower error growth than most alternatives. Second, external tests on 6 heterogeneous datasets of different sizes and library protocols indicate that the approach transfers across platforms; global similarity remains high, and expression structure among non-zero entries is preserved across datasets with different sparsity levels. A downstream plausibility check on GSE102475 further shows clearer separation of differentially expressed genes under a fixed analysis pipeline, and quantitative DE analysis demonstrates that GSCI better preserves the original DE structure than the SGM-only recovery. At the gene level, the recovery trends of key breast-cancer genes further support the biological plausibility of the reconstructed profiles.

The framework also has limitations. Performance can vary on extremely sparse, highly heterogeneous cohorts; outcomes still depend on standard preprocessing choices; and although the method is practically deployable, a more systematic profiling of runtime and memory under very large matrices would be valuable for users handling hundreds of thousands of cells. In addition, mask-augmented experiments approximate reference values without replacing fully synthetic benchmarks or spike-in controls, and our downstream analyses—while indicative—do not exhaust the breadth of single-cell tasks.

Future work will therefore focus on (a) scaling and acceleration with sparse operations and distributed training to handle larger 10x-style collections, (b) automated hyperparameter selection and uncertainty estimation, (c) broader biological validation on clustering, trajectories, and pathway enrichment across tissues and technologies, and (d) extension to multimodal and spatial assays where generative priors and sparsity constraints can be integrated more tightly. Future work will also include simulation-based benchmarking under controlled single-cell data generation settings, as well as the development of a more user-friendly software package or web-based interface to improve practical usability and reproducibility. Taken together, the evidence indicates that combining structure-guided generative recovery with sparse refinement provides an effective and biologically coherent strategy for restoring single-cell expression data.

## Data Availability

The dataset used in this article is an open-source dataset with no personal privacy or related information. The dataset used in this paper is from Gene Expression Omnibus (GEO) database, and the website is https://www.ncbi.nlm.nih.gov/geo/. The source code of GSCI is available at https://github.com/naonao0708/GSCI-1.
